# Immune regulation in Chandipura virus infection: characterization of CD4+ T regulatory cells from infected mice

**DOI:** 10.1186/1743-422X-8-259

**Published:** 2011-05-25

**Authors:** Balakrishnan Anukumar, Prajakta Shahir

**Affiliations:** 1 Chandipura Virus Group, National Institute of Virology, 20-A, Dr.Ambedkar Road, Post Box No.11. Pune-411001, India

## Abstract

**Back ground:**

Chandipura virus produces acute infection in mice. During infection drastic reduction of CD4+, CD8+ and CD19 + cell was noticed. Depletion of lymphocytes also noticed in spleen. The reduction may be due to the regulatory mechanism of immune system to prevent the bystander host tissue injury. There are several mechanisms like generation of regulatory cells, activation induced cell death (ACID) etc were indicated to control the activation and maintain cellular homeostasis. Role of regulatory cells in homeostasis has been described in several viral diseases. This study was undertaken to characterize CD4+T regulatory cells from the infected mice.

**Method:**

In this study we purified the CD4+ T cells from Chandipura virus infected susceptible Balb/c mice. CD4+ T regulatory cells were identified by expression of cell surface markers CD25, CD127 and CTLA-4 and intracellular markers Foxp3, IL-10 and TGF-beta. Antigen specificity and ability to suppress the proliferation of other lymphocytes were studied *in vitro *by purified CD4+CD25+T regulatory cells from infected mice. The proliferation was calculated by proliferation module of Flow Jo software. Expression of death receptors on regulatory cells were studied by flowcytometer.

**Results:**

The CD4+ T cells isolated from infected mice expressed characteristic markers of regulatory phenotype at all post infective hours tested. The CD4+ T regulatory cells were proliferated when stimulated with Chandipura virus antigen. The regulatory cells did not suppress the proliferation of splenocytes stimulated with anti CD3 antibody when co cultured with them. Interesting observation was, while purification of CD4+ T cells by negative selection, the population of cells negative for CD4 also co purified along with CD4+ T cell. Flow cytometry analysis and light microscopy revealed that CD4 negative cells were of different size and shape (atypical) compared to the normal lymphocytes. Greater percentage of these atypical lymphocytes expressed *Fas *Ligand and Programmed Death1 (PD-1) receptor.

**Conclusion:**

From these results we concluded that virus specific CD4+T regulatory cells are generated during Chandipura virus infection in mice and these cells might control the activated lymphocytes during infection by different mechanism.

## Background

Chandipura virus belongs to the family *Rhabdovirdae*, genus *vesiculovirus *associated with acute encephalitis and severe fatality in young children [1,2]. Children below 15 years old were vulnerable but adults were refractory to the infection. The age dependent susceptibility has also been noticed in mice [3-5]. Chandipura virus produces acute infection and induces reduction in the percentage of CD4, CD8 and CD19 positive cells in infected susceptible mice [6]. Lymphocytes reduction during acute viral infection is common in several viral diseases [7-9]. After encountering the pathogen, the lymphocytes become activated and proliferate to eliminate the pathogen. This activated T lymphocytes are continuously generated during immune response against infection and it should be removed from the immune system to prevent the bystander tissue injury as well as to maintain the cellular homeostasis. There are several mechanisms like generation of regulatory cells, activation induced cell death (ACID) etc were indicated to control the activation and maintain cellular homeostasis.

Role of regulatory cells in homeostasis have been described in several reviews [10,11]. These cells serve to limit the activation and effectors functions of CD4+ and CD8 + T cells. These cells not only protect from autoimmune disease but also protect from exogenous antigen [12]. Several proposed mechanisms of regulatory cells to limit T cell response include expression of IL-10, TGF-β, surface expression of CTLA-4 receptor, IL-2 sequestration, blockade of co stimulatory molecules etc [13]. Regulatory T cells are diverse in nature and include at least three populations that differ by their phenotype, cytokine profile and suppressive mechanism [14].

The involvement of *Fas*-*Fas *ligand in AICD was well reviewed by Stephen Maher et al [15]. As part of the host cell defense mechanism, it may reduce virus growth as well as it spread and dissemination within the organism. Recent studies have shown that the Programmed death 1 (PD-1) inhibitory pathway plays a critical role in modulating the functional exhaustion of virus specific T cells and play a dual role in immune regulation by preventing the attack on self and keeping activated immune system in check. This has now been documented in several animal models such as murine Lymphocytic Choriomeningitis virus (LCMV) infection [16] and simian immunodeficiency virus (SIV) infection of non-human primates [17] and, more importantly, in humans during persistent infection with human immunodeficiency virus (HIV), hepatitis B virus (HBV) and hepatitis C virus (HCV) [18,19].

This study is focused on whether the CD4+ T regulatory cells induced during experimentally Chandipura virus infected mice or not. These cells play an important role in cellular homeostasis. Chandipura virus specific CD4+ T regulatory cells induced during infection.

## Methods

### Cells, virus and animals

The strain 034267 was originally isolated from encephalitis outbreak in Andhra Pradesh, India, 2003. The virus was propagated and titrated in Vero E6 cells and maintained in our laboratory. The Vero E6 cells were obtained from National Center for Cell Science, India and were grown in DMEM (Pan Biotech) supplemented with 10% Fetal Calf Serum (FCS, Hyclone). Balb/c mice maintained in institute animal house were used in this study. The mice at 13 days old were used in this experiment. The mice were maintained in *adlibidum *water and feed throughout the experiment. The study was approved by institute animal ethical committee (IAEC). For experimental infection, the mice were inoculated with 10 μl (titer 5 log LD50/100 μl) of virus through footpad. Uninfected healthy mice of same age group were kept as a control throughout the study. All the assays described in this study carried out till 72 h PI because most of the infected mice died at 96 h PI.

### Purification of splenocytes and staining for Flow cytometry

Single cell suspension of splenocytes were prepared by teasing the spleen in nylon mesh containing 5 ml of RPMI 1640 supplemented with 10% FCS, 25 mM HEPES and 5 × 10^-5 ^M β-mercaptoethanol (Growth medium). The mononuclear cell population was purified by density gradient centrifugation using histopaque (1.083 gm/ml, Sigma) and washed twice in RPMI 1640 medium. The cells were counted and stained with various anti mouse antibody conjugated with different fluorescent molecules. Acquisition and analysis were done in FACScalibur using cell quest pro soft ware (BD Bioscience). In some analysis FlowJo software (Tristar) was used. The lymphocyte population was gated in forward scatter (FSC) *vs *side scatter (SSC) dot plot. The CD3 positive cells were further gated from the lymphocyte population in FSC *vs *CD3+ dot plot. CD3+CD4+ population was analyzed for expression of different markers.

### Purification of CD4+ cells from splenocytes

Splenocytes from infected as well as uninfected mice were isolated as described above. The CD4+ cells were purified from the splenocytes by using mouse CD4+ cell isolation kit (Miltenyi Biotec) as per the manufacturer's instruction. The purity was checked by staining with anti mouse CD4 antibody conjugated with fluorescein isothiocyanate (FITC) and analyzed in FACScaliber.

### Phenotypic characterization of CD4+ T cells from infected mice

#### Surface receptor and ligand expression

The purified CD4+ cells were stained with anti mouse CD4, CD25, CD127 and CTLA4 (CD152) antibody conjugated with different fluorescent molecules (eBioscience). The stained cells were analyzed in FACScaliber. The percentage of CD4+ cells expressing different markers was calculated by following formula

### Quantitation of Foxp3, TGF-β, IL-10 transcripts

Total RNA was extracted from one million CD4+ T cells using PureLink RNA mini kit (Invitrogen) according to the manufacturer's instructions. The RNA was eluted in 50 μl volume and 8 μl was used for cDNA synthesis. The level of expression of Foxp3, IL-10 and TGF-β was quantitated by SYBR green based real time PCR using published primers [13]. Two μl of cDNA was used for amplification using MESA green qPCR kit (Eurogentech). To normalize the data, Cyclin A, the housekeeping gene was amplified. Relative fold change of expression of different transcript was calculated by formula 2^- ΔΔCt^. The ΔΔCt was calculated from uninfected control.

### Expression of death receptors

The purified cells were also stained with anti mouse *Fas *(CD95), *Fas *ligand (CD95L), PD-1, TRANCE, and CD40L antibody conjugates. The stained cells were analyzed by flow cytometer as mentioned above. The percentage of CD4+ or CD4- cells positive for above mentioned receptors were calculated.

### Functional characterization of CD4+T regulatory cells

#### Purification of CD4+CD25+ regulatory T cells (T regs)

Splenocytes from infected as well as uninfected animal were purified as described above. The CD4+CD25+ T cells were purified from the splenocytes by using mouse CD4+CD25+ T regulatory cell isolation kit (Miltenyi Biotec) as per the manufacturer's instruction.

#### T reg proliferation assay

The purified T regs were labeled with carboxyfluorescein succinimidyl ester (CFSE) as per the standard procedure. Briefly, 20 million cells in one ml of PBS+0.1% BSA was stained with 5 μM final concentration of CFSE and incubated 15 min in RT. The reaction was stopped by addition of equal volume of FCS. The cells were washed twice with growth medium. The final cell pellet was seeded in to the 24 well plate containing inactivated Chandipura virus whole antigen pulsed splenic macrophages. T regs from control mice was also treated in the same way. The cells were incubated for three days at 37°C in 5% CO_2_. After incubation, the cells were stained with anti mouse CD4-PE-Cy7 and acquired in FACScaliber and analyzed in Flow Jo software using proliferation module.

### Suppression assay

Splenocytes from normal mice were labeled with CFSE as described above. The labeled splenocytes were co cultured with T regs from infected mice in the presence of 1 μg/ml of mouse anti CD3 antibody (eBioscience). Appropriate control including only splenocytes and splenocytes with anti CD3 antibody also kept in the plate. The cells were incubated for five days at 37°C in 5% CO_2_. After incubation the cells were stained with anti mouse CD4-PE-Cy7 conjugate and acquired in flow cytometer. The acquired data was analyzed by flow Jo soft ware using proliferation module.

### Statistical analysis

Student's *t *test was used to compare the treated and control groups. The *p *value less than 5% was considered as significant.

## Results

### Induction of CD4+T regulatory cells during infection

**Figure 1:**
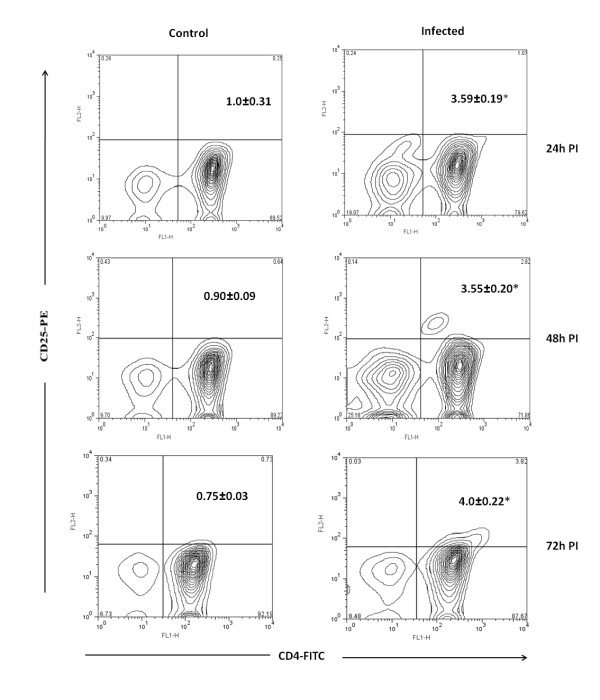
**Expression of CD25 on CD4+T cells from mice**. The CD4+ T cells from infected and control mice were purified at different hours post infection (PI) and stained with anti mouse CD25 antibody conjugated with phycoerythrin (PE). The percentage of CD4+ T cells positive for CD25 were calculated by formula mentioned in methods. The biexponential transformation of dot plot represents the percentage of CD4+ population which showed significant changes in expression of CD25 receptor at different hour post infection. Values in upper right corner are Mean ± SE of three independent experiments,*p < 0.01

**Figure 2:**
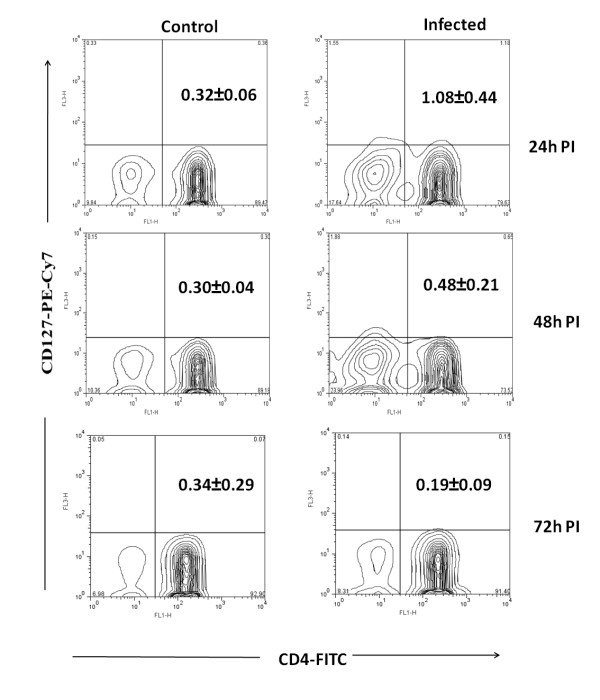
**Expression of CD127 on CD4+T cells from mice**. The CD4+ T cells from infected and control mice were purified at different hours post infection (PI) and stained with anti mouse 127 antibody conjugated with phycoerythrin-Cy7 (PE-Cy7). The percentage of CD4+ T cells positive for CD127 were calculated by formula mentioned in methods. The biexponential transformation of dot plot represents the percentage of CD4+ population which showed expression of CD127 receptor at different hour post infection. Values in upper right corner are Mean ± SE of three independent experiments.

**Figure 3:**
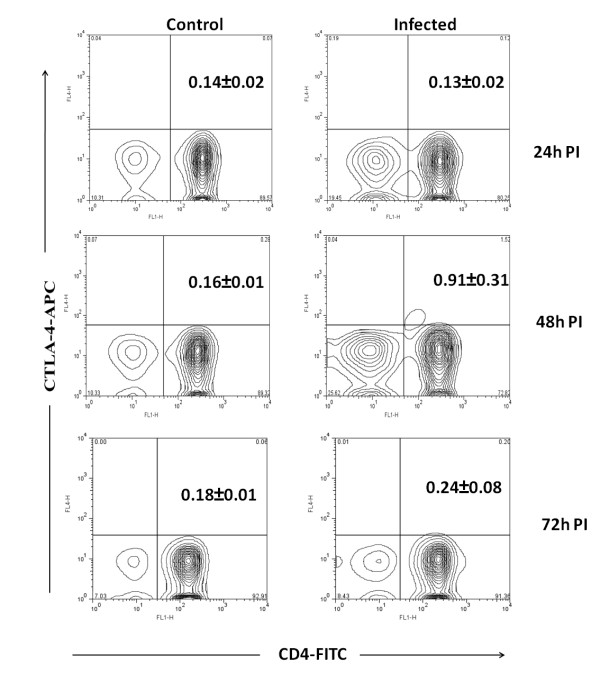
**Expression of CTLA-4 on CD4+T cells from mice**. The CD4+ T cells from infected and control mice were purified at different hours post infection (PI) and stained with anti mouse CTLA-4 antibody conjugated with allo-phycocyanin (APC). The percentage of CD4+ T cells positive for CTLA-4 were calculated by formula mentioned in methods. The biexponential transformation of dot plot represents the percentage of CD4+ population which showed expression of CTLA-4 receptor at different hour post infection. Values in upper right corner are Mean ± SE of three independent experiments.

**Figure 4:**
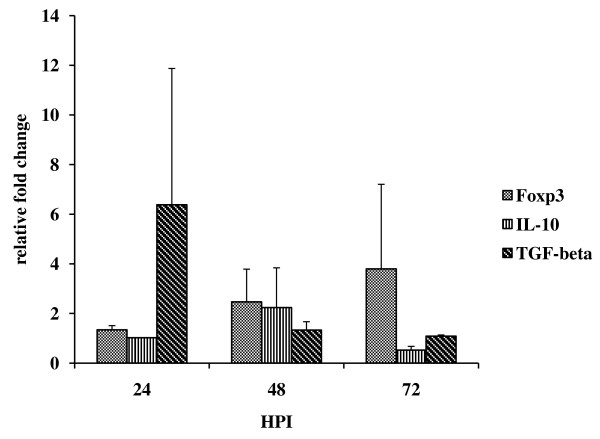
**Expression of Foxp3, IL-10 and TGF-β transcripts in CD4+T cells from infected mice**. The CD4+ T cells from infected and control mice were purified at different hours post infection (HPI) and the expression of Foxp3, IL-10 and TGF-β transcripts were quantitated by SYBR green based real time RT-PCR. The relative fold change (two fold) was calculated from uninfected control as mentioned in methods. The values are Mean ± SE of three independent experiments.

The CD4+ T regulatory cells can be differentiated from CD4+T cells by level of expression of CD25 and CD127 on cell surface. Moreover these cells also expresses the transcription factor Foxp3 and the cytokines IL-10 and TGF-β. These cytokines play a major role in regulation of immune system. In Chandipura infected mice CD4+CD25+ T cells were noticed from 24 h PI onwards and the level was significantly high at 72 h PI (4%, *p* < 0.01) (Figure 1). The CD4+CD127+T cell level was decreased from 24 h PI onwards and it was lower than control at 72 h PI (Figure 2). No significant difference was noticed in CTLA-4 expression (Figure 3). The level of expression of Foxp3, IL-10 and TGF-β was quantitated by SYBR green based real time RT-PCR. The result indicated that the expression of these transcripts were noticed in all PI hours tested (Figure 4).

### Antigen specificity of T regs

**Figure 5:**
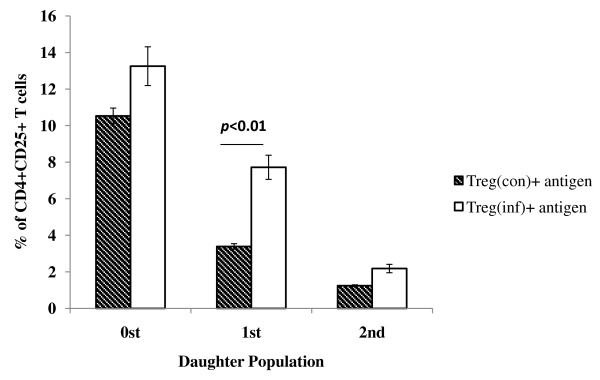
**In vitro Chandipura antigen specific proliferation of CD4+CD25+ T cells (T regs) from infected and control mice**. The double positive cells were purified and stained with CFSE and co cultured with splenic macrophages pulsed with Chandipura antigen. The proliferation was calculated at 72 h post infection by proliferation module in Flow Jo software. It is evident that proliferation *in vitro *because their level of the CFSE dye, which is divided equally among daughter, cells upon cell division has decreased. The percentage of CD4+CD25+T cells in different daughter population was calculated. The values are Mean ± SE of triplicate.

T regs cells isolated from both control and infected mice were stimulated with inactivated whole Chandipura virus antigen. Proliferation was calculated by Flow Jo software. It is evident that proliferation of CD4+CD25+T cells *in vitro *because their level of the CFSE dye, which is divided equally among daughter cells upon cell division, has decreased. The percentage of CD4+CD25+T cells in different daughter population was calculated. The percentage of CD4+CD25+ T cells from infected mice was 7.72% in first generation daughter population and it was 3.39% in control mice (*p* < 0.001). Similarly in second generation daughter cell it was 2.18% in infected and 1.24% in control (Figure 5).

### Suppression of proliferation of CD4+T cells by T regs

**Figure 6:**
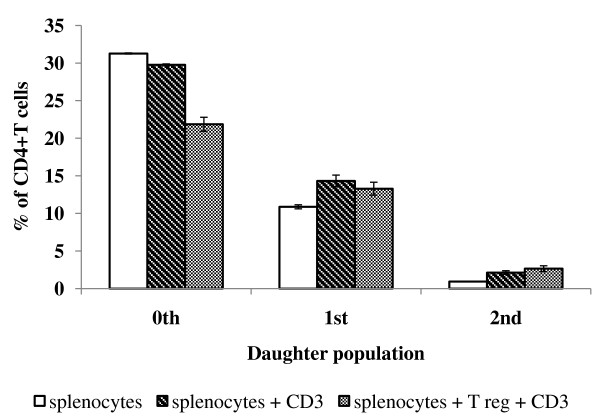
**Proliferation of anti CD3 antibody stimulated splenocytes in the presence of CD4+CD25+ T cells (T regs) from infected mice**. The splenocytes were stained with CFSE and the proliferation was calculated on 5^th ^day post stimulation by proliferation module. The values are Mean ± SE of triplicate.

No significant suppression was noticed in anti CD3 antibody stimulated splenocytes from normal mice co cultured with T regs from infected mice (Figure 6).

### CD4 negative population in infected mice

**Figure 7:**
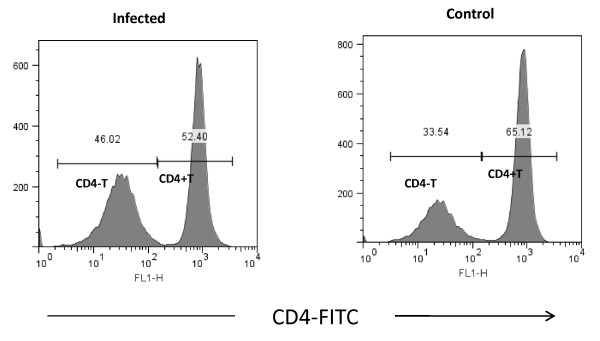
**Percentage of CD4 negative population of cells which was co purified along with CD4+ cells purified by negative selection from infected and control mice**. In histogram CD4-T marker represents the percentage of cells unstained with mouse anti CD4 antibody and CD4+T marker represents percentage of cells stained with mouse anti CD4 antibody.

**Figure 8:**
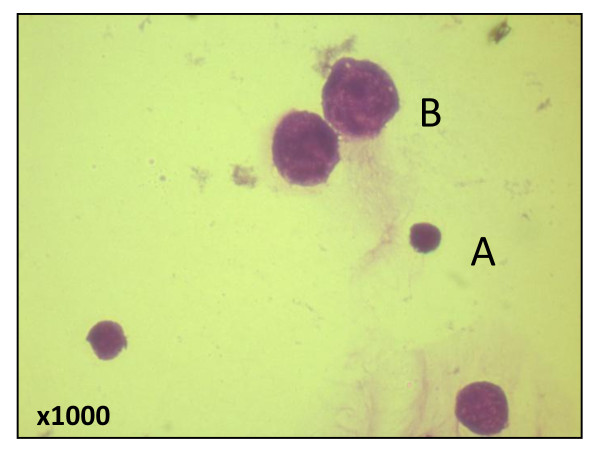
**Leishman's staining of purified CD4+ T cells from infected mice**. A. normal lymphocytes B. large size lymphocytes with dispersed chromatin.

**Figure 9:**
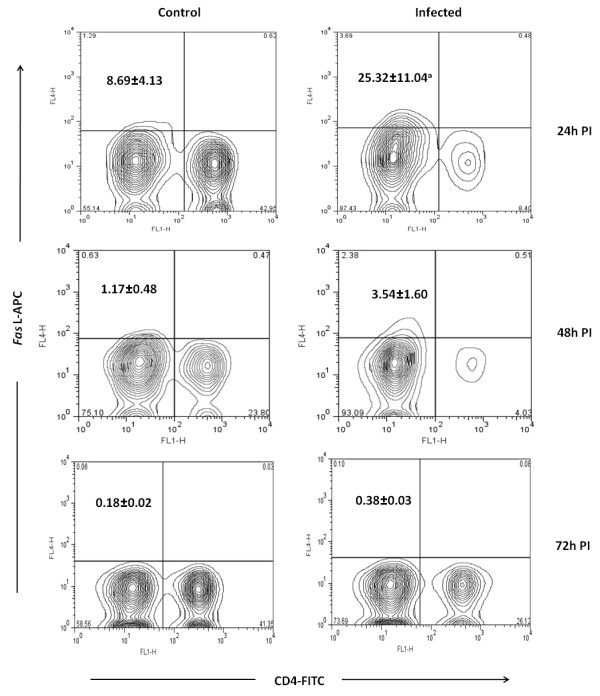
**Expression of *Fas *ligand on CD4 negative T cell from mice**. The CD4+ T cells from infected and control mice were purified at different hours post infection (PI). The cells were stained with anti mouse *Fas *ligand antibody conjugated with allo-phycocyanin (APC). The cells negative for CD4 receptors were gated and the percentage of CD4 negative T cells expressed the *Fas *ligand were calculated by formula mentioned in methods. The biexponential transformation of dot plot represents the percentage of CD4- population which showed significant changes in expression of *Fas *ligand at different hour post infection (PI). ^a ^*p *< 0.05

**Figure 10:**
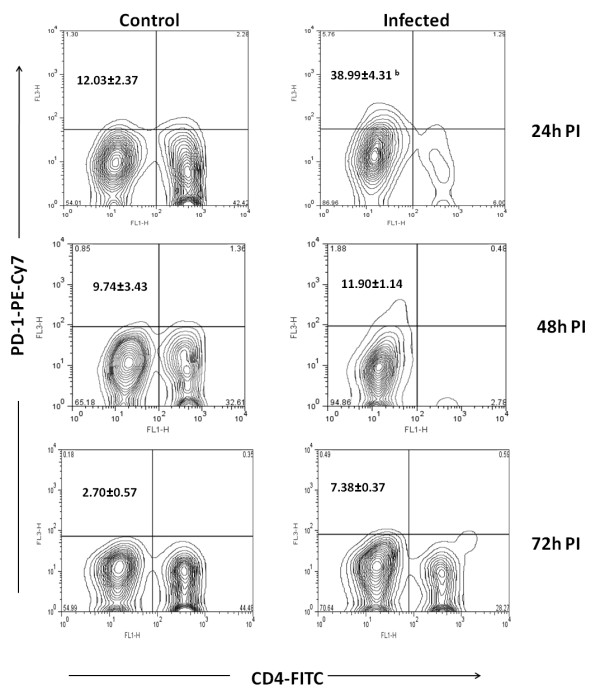
**Expression of Programmed Death 1 (PD-1) on CD4 negative T cell from mice**. The CD4+ T cells from infected and control mice were purified at different hours post infection (PI). The cells were stained with anti mouse PD-1 antibody conjugated with phycoerythrin-Cy7 (PE-Cy7). The cells negative for CD4 receptors were gated and the percentage of CD4 negative T cells expressed the PD-1 were calculated by formula mentioned in methods. The biexponential transformation of dot plot represents the percentage of CD4- population which showed significant changes in expression of PD-1 at different hour post infection (PI).^b ^*p *< 0.01

While purification of CD4+T cells by negative selection, cells negative for CD8, CD11b, CD45R, CD49b and Ter-119 also purified along with CD4+Tcells. The CD4- population was approximately 10% more in infected mice (Figure 7). Leishman's staining of this cell showed different morphology with distributed chromatin (Figure 8). Both the CD4+ and CD4- cells were stained with *Fas*, *Fas *ligand, PD-1, CD40L, TRANCE and CD69 markers. Approximately 25% of CD4- cells expressed *Fas *ligand compared to CD4- cells from control (8%) at 24 h PI (*p *< 0.05) (Figure 9). The difference in expression was also noticed at 48 h PI. Similarly 38% of this cell also expressed PD-1 receptor and in control it was 12% (*p *< 0.01). The PD-1 receptor expression was noticed in entire PI hours tested (Figure 10). No significant changes between infected and control was noticed in other receptors tested in this experiment. Similarly no significant changes in expression were noticed in these receptors in CD4+ cells also (data not shown).

## Discussion

Silencing of T cell response to acute viral infection is essential to maintain the homeostasis of immune cells and also to avoid the untoward effects on bystander cells. Because of this reason, several acute viral infections produces lymphocyte reduction in host [7-9]. In Influenza A infection in human it was reported that virus replication in lymphocytes leads to reduction of lymphocytes [7]. In this study the mechanism behind the reduction was analyzed with focus on T regulatory cells.

The CD4+T cells from infected mice expressed CD25, Foxp3+, IL-10 and TGF-β, the characteristic markers of regulatory phenotype of T cells. This regulatory population of cells noticed from 24 h PI onwards and highest number of population noticed at 48 and 72 h PI. Chandipura viral antigen specific proliferation of these cells indicated that these cells are virus specific. These cells were activated during infection but did not suppress the proliferation of CD3 stimulated normal splenocytes *in vitro*.

Interesting observation during purification of CD4+ cell was that the cells negative for CD4 and all other markers used in depletion was co purified along with CD4+ cells. Morphologically these cells were larger than lymphocytes and showed various size and shape (atypical). The chromatin was dispersed throughout the cells. These cells are called as atypical or reactive lymphocytes and described by Simon (2003) [20] in his review. The greater percentage of these cells expressed PD-1 receptor and *Fas *ligand. *Fas *L is a death ligand induces apoptosis in cell that express *Fas *receptor. Similarly PD-1 is extended family of CD28/CTLA-4 present on T cell regulators [21,22] which negatively regulates the TCR signals. It is now known that up regulation PD-1 in exhausted CD8 T cells is coincident with the progression of many chronic human diseases including human immunodeficiency virus (HIV), hepatitis C virus, (HCV), and Epstein Barr virus (EBV) [17,18,23]. Greater percentage of PD-1 expression in acute Chandipura virus infection indicated that immune system might suppress the activated lymphocytes through PD-1 and PD-1 ligand interaction to prevent bystander tissue injury. The suppression of T cells might allow the infection to progress because most of the infected mice died at 96 h PI.

## Conclusion

This study concluded that different regulatory mechanisms activated during Chandipura virus infection in mice. The induction of CD4+T regulatory cells and expression of PD-1 may be one of the mechanisms by which mice immune system control the activated lymphocytes and maintain the homeostasis. The exact role of these cells in immune regulation needs to be studied.

## Competing interests

The authors declare that they have no competing interests.

## Authors' contributions

BA designed the study, performed the experiments and drafted the manuscript. PS helped to do some of the experiments. All the authors read and approved the final manuscript.
